# Supplementary material for: A high-resolution mRNA expression time course of embryonic development in zebrafish
Source: eLife. 2017 Nov 16;6:e30860. doi: 10.7554/eLife.30860 (PMC5690287; doi:10.7554/eLife.30860)
Supplement: Supplementary file 6. [file elife-30860-supp6.zip › biolayout-clusters-files/Cluster113-genes.html]

Cluster113


# Cluster113: Genes

| Ensembl ID | Gene Name | Chr | Start | End | Biotype |
| --- | --- | --- | --- | --- | --- |
| ENSDARG00000101220 | POLRMT | 22 | 18993425 | 19077396 | protein\_coding |
| ENSDARG00000038866 | TRAPPC8 | 20 | 6263722 | 6486869 | protein\_coding |
| ENSDARG00000079672 | eftud1 | 7 | 11887994 | 12118245 | protein\_coding |
| ENSDARG00000062532 | fam21c | 13 | 17942170 | 17991350 | protein\_coding |
| ENSDARG00000033604 | lrpap1 | 1 | 40428694 | 40438659 | protein\_coding |
| ENSDARG00000036541 | rhbdf1a | 3 | 54641715 | 54738425 | protein\_coding |
| ENSDARG00000016336 | tmco6 | 14 | 8150756 | 8166279 | protein\_coding |
| ENSDARG00000044513 | tpst1l | 21 | 31196178 | 31204559 | protein\_coding |
| ENSDARG00000074435 | ttc19 | 2 | 32492576 | 32502862 | protein\_coding |
| ENSDARG00000008224 | vps35 | 7 | 41477391 | 41532282 | protein\_coding |
| ENSDARG00000025346 | znf277 | 25 | 21618715 | 21646374 | protein\_coding |
